# Early Predictors of In-Hospital Mortality and Cardiac Dysfunction in Patients with ST-Segment Elevation Myocardial Infarction Undergoing Early Revascularization

**DOI:** 10.3390/jcm15093256

**Published:** 2026-04-24

**Authors:** Corina Cinezan, Alexandra Manuela Buzle, Camelia Bianca Rus

**Affiliations:** 1 Department of Medical Disciplines, Faculty of Medicine and Pharmacy, University of Oradea, 410073 Oradea, Romania; buzle.alexandramanuela@student.uoradea.ro (A.M.B.); rus.cameliabianca@student.uoradea.ro (C.B.R.); 2 Clinical County Emergency Hospital Bihor, 410169 Oradea, Romania; 3 Doctoral School of Biological and Biomedical Sciences, University of Oradea, 410087 Oradea, Romania

**Keywords:** ST-segment elevation myocardial infarction, primary percutaneous coronary intervention, in-hospital mortality, left ventricular ejection fraction, IMR, risk predictors, complete revascularization

## Abstract

**Background:** Despite advances in reperfusion therapy, ST-segment elevation myocardial infarction (STEMI) remains associated with substantial morbidity and mortality. Early identification of predictors of adverse outcomes is essential for improving risk stratification. **Methods**: This retrospective study included 512 STEMI patients who underwent coronary revascularization within 6 h of symptom onset. Clinical, laboratory, angiographic and echocardiographic variables were analyzed. The primary endpoint was in-hospital mortality. Secondary outcomes included reduced left ventricular ejection fraction (LVEF < 40%) and moderate-to-severe ischemic mitral regurgitation (IMR). Independent predictors of in-hospital mortality were identified using multivariable logistic regression, while secondary outcomes were described to characterize the study population. Model performance was evaluated using ROC analysis. **Results**: In-hospital mortality occurred in 9.4% of patients. Reduced LVEF was present in 26.2%, and IMR in 10.9%. Independent predictors of mortality included LVEF < 40% (OR 5.72, 95% CI 2.77–11.80, *p* < 0.001), IMR (OR 2.61, 95% CI 1.14–5.97, *p* = 0.023), lower hemoglobin levels (OR 0.74, 95% CI 0.61–0.91, *p* = 0.003), and reduced glomerular filtration rate (OR 0.96, 95% CI 0.95–0.98, *p* < 0.001). The model demonstrated good discrimination (AUC 0.88). Complete revascularization was not independently associated with mortality. **Conclusions**: Left ventricular dysfunction, IMR, anemia, and renal impairment are strong predictors of in-hospital mortality in STEMI patients. Integrating echocardiographic and laboratory parameters may improve early risk stratification and guide clinical decision-making.

## 1. Introduction

ST-segment elevation myocardial infarction continues to be a major cause of cardiovascular morbidity and mortality worldwide, despite a significant advance in early diagnosis and reperfusion strategies [1]. Prompt re-establishment of coronary blood flow through primary percutaneous coronary intervention (PCI) has substantially improved survival in patients presenting with acute myocardial infarction [2]. Nevertheless, a considerable proportion of patients continue to experience adverse outcomes, including in-hospital mortality, left ventricular systolic dysfunction and IMR [3]. The identification of predictors of these complications is therefore essential for early risk stratification and optimal management of STEMI patients.

One of the most important determinants of prognosis after acute myocardial infarction remains left ventricular systolic dysfunction. Reduced left ventricular ejection fraction reflects the extent of myocardial injury and is related to an increased risk of heart failure, arrhythmias and mortality [4]. In addition to ventricular dysfunction, structural complications such as IMR may occur following myocardial infarction as a consequence of papillary muscle displacement, ventricular remodeling and altered leaflet cooptation [5]. A moderate or severe IMR has been associated with worse clinical outcomes and increased mortality in several observational studies [6].

Besides echocardiographic parameters, clinical and laboratory variables may also contribute to risk stratification in patients with STEMI. Factors such as anemia, renal dysfunction and inflammatory markers have been reported to influence outcomes in acute coronary syndromes [7]. Renal impairment, frequently observed in patients with cardiovascular disease, is associated with systemic inflammation, endothelial dysfunction and a higher burden of comorbidities [8]. Similarly, lower hemoglobin levels may aggravate myocardial ischemia by reducing oxygen delivery to the injured myocardium [9].

In contemporary clinical practice, comprehensive evaluation of STEMI patients includes the integration of clinical, laboratory, angiographic and echocardiographic information [1]. However, the relative contribution of these factors to adverse outcomes may vary across patient populations and healthcare settings. Therefore, further investigation of predictors of mortality and cardiac dysfunction remains important for improving risk stratification models.

In addition, the extent of coronary revascularization, particularly complete versus incomplete revascularization, has been shown to influence clinical outcomes in patients with STEMI, although its impact on early in-hospital prognosis remains incompletely defined [10].

The primary aim of the present study was to identify factors associated with in-hospital mortality in patients presenting with ST-segment elevation myocardial infarction who underwent coronary revascularization within 6 h of symptom onset. Secondary outcomes, including reduced left ventricular ejection fraction (LVEF < 40%) and the presence of moderate or severe IMR, were evaluated descriptively to better characterize the clinical profile of the study population.

## 2. Materials and Methods

### 2.1. Study Design and Population

This study represents a retrospective observational analysis of 512 consecutive adult patients with STEMI admitted between January 2021 and August 2025 in the Cardiology Department of Clinical County Emergency Hospital Bihor. All patients underwent coronary revascularization within the first 6 h after symptom onset. The study aimed to identify early predictors of adverse in-hospital outcomes, including mortality, reduced left ventricular ejection fraction (LVEF < 40%) and moderate or severe IMR.

### 2.2. Inclusion Criteria

Patients were eligible for inclusion if they met the following criteria: (1) diagnosis of ST-segment elevation myocardial infarction according to contemporary international guidelines; (2) presentation within 6 h from symptom onset; (3) treatment with coronary revascularization, primarily primary percutaneous coronary intervention; and (4) availability of complete clinical, laboratory, and echocardiographic data required for the analysis.

### 2.3. Exclusion Criteria

Patients were excluded from the analysis if they met any of the following criteria: (1) missing essential clinical or laboratory data required for statistical analysis; (2) absence of echocardiographic evaluation during the index hospitalization; (3) previous severe structural valvular disease unrelated to IMR; (4) presentation beyond 6 h after symptom onset; or (5) incomplete follow-up regarding in-hospital outcomes.

### 2.4. Clinical and Echocardiographic Evaluation

Baseline demographic and clinical data were collected from the study dataset, including age, sex, cardiovascular risk factors (smoking, diabetes mellitus, hypertension, dyslipidemia, and obesity), and infarction localization (anterior, inferior, lateral, posterior, or right ventricular involvement).

Echocardiographic evaluation was performed during the index hospitalization using standard transthoracic echocardiography. Left ventricular ejection fraction (LVEF) was assessed using standard echocardiographic methods and expressed as a percentage. Reduced systolic function was defined as LVEF < 40%. IMR was evaluated using Doppler echocardiography and classified according to standard grading criteria. For the purposes of this study, IMR was defined as moderate or severe mitral regurgitation [11,12].

Laboratory parameters obtained at admission included hemoglobin levels, white blood cell count, neutrophils, lymphocytes, red cell distribution width, mean platelet volume, platelet count, serum creatinine, and estimated glomerular filtration rate (GFR). These variables were evaluated as potential predictors of adverse outcomes.

### 2.5. Angiographic Evaluation and Treatment

Coronary angiography was performed in all patients during the index hospitalization as part of the primary percutaneous coronary intervention strategy for the treatment of ST-segment elevation myocardial infarction. Coronary thrombotic occlusion of the infarct-related artery was defined as the presence of a complete or near-complete interruption of antegrade coronary blood flow caused by an intraluminal thrombus, typically corresponding to Thrombolysis in Myocardial Infarction (TIMI) flow grade 0–1 on angiographic assessment.

Significant coronary artery stenosis requiring PCI was defined as the presence of a luminal diameter reduction ≥70% in a major epicardial coronary artery or ≥50% stenosis in the left main coronary artery, as visually estimated by coronary angiography. In the context of acute STEMI, lesions considered responsible for the infarction (culprit lesions) were treated with primary PCI regardless of the exact degree of stenosis when angiographic findings and clinical presentation indicated an acute thrombotic event.

Multivessel coronary artery disease was defined as the presence of significant stenosis in two or more major epicardial coronary arteries. Complete revascularization was defined as treatment of all angiographic ally significant lesions either during the index procedure or during the same hospitalization. Given the observational design, this definition may introduce time-dependent (immortal-time) bias, as patients must survive long enough to undergo staged procedures.

All patients received in-hospital medical treatment according to guidelines.

### 2.6. Outcome Measures

The primary outcome of interest was in-hospital mortality. Secondary outcomes included the presence of reduced left ventricular systolic function (LVEF <40%) and moderate or severe IMR detected during hospitalization.

### 2.7. Statistical Analysis

Continuous variables were expressed as mean ± standard deviation, while categorical variables were presented as frequencies and percentages. Group comparisons were performed using Student’s *t*-test or Mann–Whitney U-test for continuous variables depending on distribution, and the chi-square test or Fisher’s exact test for categorical variables.

To identify predictors of in-hospital mortality, univariable logistic regression analysis was first performed for potential clinical, laboratory, and echocardiographic variables.

Variables considered clinically relevant were included in the multivariable model. Univariable logistic regression analyses were performed to explore associations between candidate variables and in-hospital mortality and to support model development.

The results of logistic regression analyses were expressed as odds ratios (ORs) with 95% confidence intervals (CIs). The discriminatory ability of the mortality prediction model was assessed using receiver operating characteristic (ROC) curve analysis and the area under the curve (AUC). Model calibration was evaluated using calibration plots comparing predicted and observed outcomes. Collinearity between variables included in the multivariable model was assessed, and no significant multicollinearity affecting model stability was identified.

Complete revascularization was included as a binary variable in the regression models.

A two-sided *p*-value < 0.05 was considered statistically significant. Statistical analyses were performed using standard statistical software.

## 3. Results

A total of 512 patients with STEMI who underwent coronary revascularization within 6 h of symptom onset were included in the analysis. The baseline demographic and clinical characteristics of the study population are presented in Table 1. The mean age of the cohort was approximately 62 years, and approximately one third of the patients were female. Cardiovascular risk factors were common, including hypertension, diabetes mellitus, smoking, and dyslipidemia. Nearly half of the infarctions were anterior, while inferior infarctions accounted for a similar proportion of cases. Complete coronary revascularization during the index hospitalization was achieved in the majority of patients.

The overall in-hospital outcomes are summarized in Table 2. In-hospital mortality occurred in 48 patients (9.4%). Reduced left ventricular systolic function, defined as left ventricular ejection fraction (LVEF) below 40%, was observed in 134 patients (26.2%). Moderate or severe IMR was identified in 56 patients (10.9%) during the index hospitalization.

### 3.1. Impact of Revascularization Strategy on Clinical Outcomes

Outcomes according to revascularization strategy are summarized in Table 3. Patients who underwent complete revascularization had lower rates of in-hospital mortality compared with those with incomplete revascularization (7.0% vs. 16.9%, *p* = 0.002).

Reduced left ventricular ejection fraction (LVEF <40%) was also less frequent among patients with complete revascularization (21.6% vs. 40.3%, *p* < 0.001).

In contrast, moderate or severe IMR was more frequent in the incomplete revascularization group compared with the complete revascularization group (16.1% vs. 9.3%, *p* = 0.046).

However, after adjustment for echocardiographic and laboratory variables, complete revascularization was not independently associated with in-hospital mortality in the multivariable model.

Laboratory and echocardiographic parameters are summarized in Table 4. The mean LVEF in the study population was approximately 43%, indicating that a significant proportion of patients presented with impaired ventricular systolic function following STEMI. Hemoglobin levels, renal function markers, and inflammatory indices demonstrated variability within the cohort, reflecting the heterogeneous clinical presentation of patients with acute myocardial infarction.

### 3.2. Multivariable Logistic Regression

Multivariable logistic regression analysis shown in Table 5 identified reduced left ventricular ejection fraction (LVEF < 40%) as the strongest independent predictor of in-hospital mortality (OR 5.72, 95% CI 2.77–11.80, *p* < 0.001). Moderate or severe IMR was also independently associated with increased mortality risk (OR 2.61, 95% CI 1.14–5.97, *p* = 0.023). Lower hemoglobin levels (OR 0.74, 95% CI 0.61–0.91, *p* = 0.003) and reduced glomerular filtration rate (OR 0.96, 95% CI 0.95–0.98, *p* < 0.001) were significantly associated with adverse outcomes. Complete revascularization was associated with a lower risk of in-hospital mortality (OR 0.65, 95% CI 0.31–1.34), although this association did not reach statistical significance after multivariable adjustment (*p* = 0.240).

**Figure 1:**
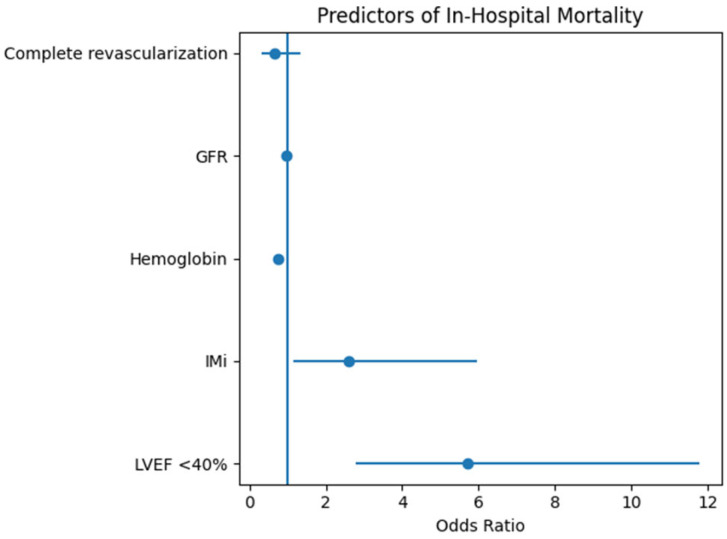
Forest plot of predictors of in-hospital mortality. LVEF = left ventricular ejection fraction; IMi = IMR; GFR = glomerular filtration rate. Complete revascularization was defined as treatment of all significant coronary lesions. Odds ratios greater than 1 indicate increased risk, while values below 1 indicate a protective association.

The results of the multivariable regression analysis are visually summarized in the forest plot shown in Figure 1. The forest plot illustrates the odds ratios and 95% confidence intervals for independent predictors of in-hospital mortality in patients with ST-segment elevation myocardial infarction undergoing early revascularization. The vertical reference line represents OR = 1. Variables with confidence intervals that do not cross 1 are considered statistically significant predictors.

Reduced LVEF and IMR demonstrated the strongest associations with mortality risk, while lower hemoglobin and decreased GFR also contributed significantly to risk stratification.

**Figure 2:**
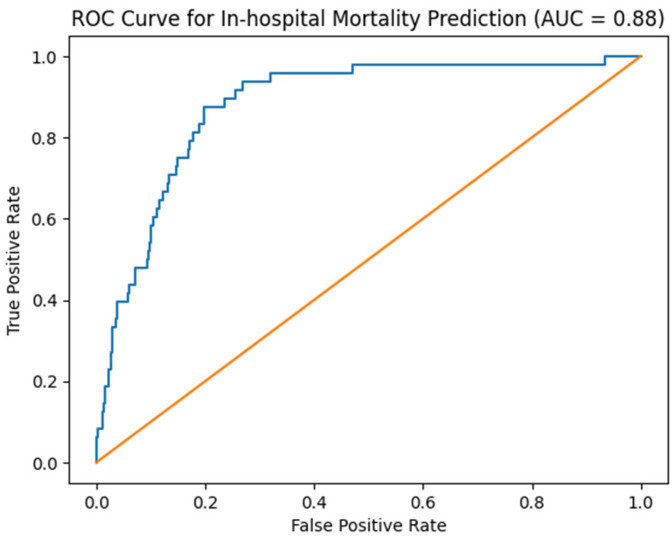
ROC curve for mortality prediction model. Area under the curve (AUC): 0.88. The area under the curve (AUC) quantifies the overall predictive performance of the model. An AUC of 0.5 indicates no discrimination, while an AUC of 1.0 indicates perfect prediction.

The discriminatory performance of the mortality prediction model is illustrated by the receiver operating characteristic (ROC) curve shown in Figure 2. The area under the curve (AUC) indicates good overall discrimination of the model for predicting in-hospital mortality.

**Figure 3:**
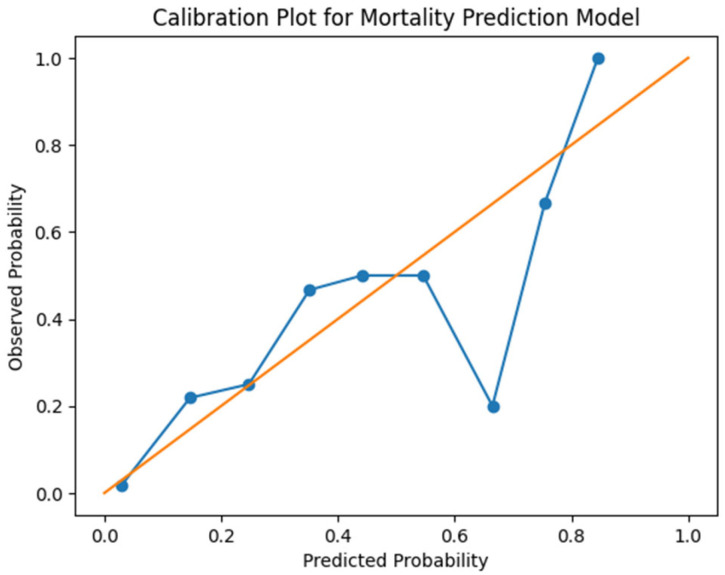
Calibration plot of the mortality prediction model. A perfectly calibrated model would follow the 45-degree reference line, indicating agreement between predicted and observed outcomes.

The calibration of the prediction model was evaluated using a calibration plot (Figure 3), which compares predicted mortality probabilities with observed event rates across risk strata. The calibration curve demonstrated a reasonable agreement between predicted and observed outcomes, supporting the reliability of the model.

**Figure 4:**
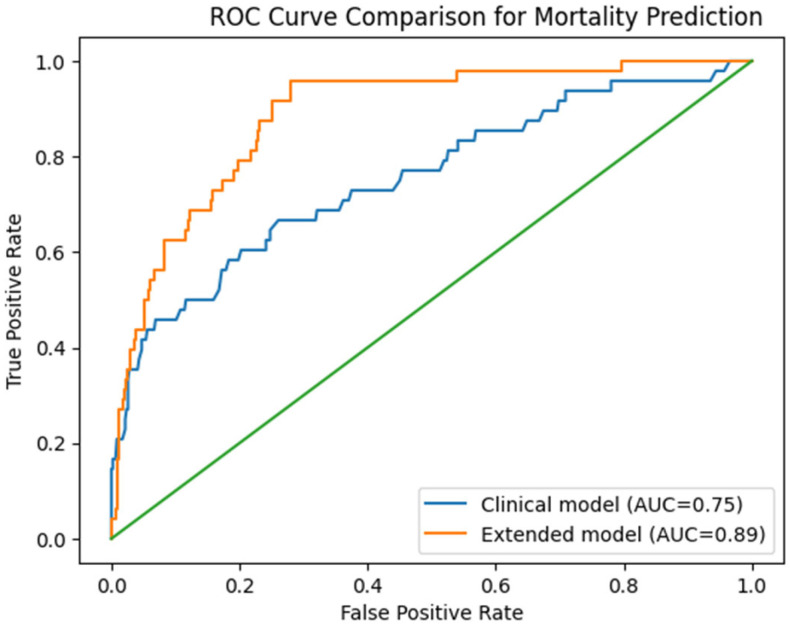
ROC curve comparison between a clinical model (age, sex, diabetes, hypertension) and an extended model including echocardiographic and laboratory variables (LVEF < 40%, IMR, hemoglobin, and GFR) for prediction of in-hospital mortality. The extended model demonstrates improved discrimination, reflected by a higher area under the curve (AUC).

A comparison of ROC curves between a clinical prediction model and an extended model incorporating echocardiographic and laboratory variables is shown in Figure 4. The extended model demonstrated improved discrimination compared with the clinical model alone, suggesting that the addition of echocardiographic and laboratory parameters enhances risk prediction in patients with STEMI undergoing early revascularization.

A formal statistical comparison between the AUCs of the two models (e.g., DeLong test) was not performed.

## 4. Discussion

This study evaluated predictors of adverse outcomes in a cohort of patients who presented with ST-segment elevation myocardial infarction and underwent coronary revascularization within the first 6 h after symptom onset. The main findings of the present analysis are that reduced left ventricular ejection fraction (LVEF < 40%), moderate or severe IMR, lower hemoglobin levels and impaired renal function were independently associated with in-hospital mortality. These findings emphasize the importance of early echocardiographic and laboratory assessment for risk stratification in STEMI patients treated with early reperfusion.

### 4.1. Interpretation of Main Findings

Reduced left ventricular systolic function emerged as the strongest predictor of in-hospital mortality in the present cohort. This observation is consistent with the well-established role of left ventricular dysfunction as a key determinant of short- and long-term prognosis following acute myocardial infarction. Reduced LVEF reflects the extent of myocardial damage and is closely associated with hemodynamic compromise, arrhythmias, and the development of heart failure.

Moderate or severe IMR was also independently associated with increased mortality risk. IMR commonly develops after myocardial infarction due to papillary muscle displacement, ventricular remodeling, and leaflet tethering. The presence of IMR reflects more extensive myocardial injury and adverse ventricular geometry, which may further worsen hemodynamic status and contribute to poorer clinical outcomes.

Lower hemoglobin levels were associated with increased mortality risk in our study. Anemia may worsen myocardial ischemia by reducing oxygen delivery to the injured myocardium and has been previously associated with adverse outcomes in acute coronary syndromes. Similarly, impaired renal function was independently associated with increased mortality. Reduced glomerular filtration rate reflects systemic vascular disease and is frequently associated with inflammation, endothelial dysfunction and increased cardiovascular risk.

### 4.2. Impact of Revascularization Strategy

In the present study, complete coronary revascularization was associated with improved crude outcomes, including lower rates of in-hospital mortality and left ventricular dysfunction. However, this association did not remain statistically significant after adjustment for key prognostic factors such as left ventricular systolic function, IMR, hemoglobin levels, and renal function. The lack of independent association between complete revascularization and in-hospital mortality likely reflects confounding by baseline disease severity. Patients undergoing complete revascularization may have had more favorable clinical profiles, including better ventricular function and less systemic comorbidity. After adjustment for these factors, the apparent benefit of complete revascularization was attenuated, suggesting that early mortality is primarily driven by myocardial injury and systemic condition rather than the extent of revascularization itself.

These findings suggest that the apparent benefit of complete revascularization may be partly mediated by its association with less severe myocardial injury and better baseline clinical status.

### 4.3. Comparison with Previous Studies

The findings of the present study are consistent with a large body of evidence identifying left ventricular systolic dysfunction as a key determinant of prognosis in patients with STEMI. The prognostic value of reduced LVEF has been extensively demonstrated in major registries and clinical trials. The GRACE study [7], a large multinational registry, showed that markers of ventricular dysfunction were among the strongest predictors of 6-month mortality. Similarly, the TIMI risk score for STEMI [13], derived from the InTIME II trial population, incorporates clinical surrogates of ventricular dysfunction and has been widely validated for early risk stratification.

In addition, analyses from large heart failure trials, such as the study by [14], demonstrated an ongoing connection between decreasing LVEF and increasing risk of cardiovascular mortality across a broad spectrum of patients. A meta-analysis of chronic heart failure trials further confirmed that LVEF remains an independent predictor of mortality even after adjustment for multiple clinical variables [15]. More recently, the VALIANT trial demonstrated that post-infarction patients with reduced LVEF had significantly higher mortality and heart failure rates despite optimal medical therapy [16]. Similarly, data from the HORIZONS-AMI trial showed that impaired LVEF after primary PCI is strongly associated with early and late mortality [17]. Furthermore, another trial provided additional evidence that early mechanical reperfusion improves outcomes, but residual ventricular dysfunction remains a major determinant of prognosis [18]. These findings align closely with the present study, in which LVEF <40% emerged as the strongest independent predictor of in-hospital mortality.

IMR has also been consistently associated with adverse outcomes following myocardial infarction. A recent study by [6] demonstrated that the presence and severity of IMR were independently associated with reduced long-term survival. More recently, the meta-analysis by [19], which included over 5000 patients, showed that even mild secondary mitral regurgitation is associated with increased mortality and heart failure hospitalization. In addition, studies such as that by [20] based on a community cohort demonstrated that IMR is common after myocardial infarction and independently predicts heart failure and death. More recently, data from Effective Regurgitant Orifice studies emphasized the quantitative relationship between regurgitation severity and survival [21]. These findings reinforce the results of the present study and highlight the importance of systematic echocardiographic evaluation early after reperfusion.

Renal dysfunction is another well-established predictor of poor outcomes in cardiovascular disease. The study by [8] which included more than one million patients, demonstrated a graded association between declining glomerular filtration rate (GFR) and increased risks of death and cardiovascular events. Another research by [22] further showed that both baseline renal impairment and worsening renal function are strongly associated with mortality. Additional large registry data, such as the SWEDEHEART registry [23], confirmed that even mild renal dysfunction significantly increases mortality risk in myocardial infarction patients treated with contemporary therapies. These findings are consistent with the present analysis, emphasizing the importance of renal function in early risk assessment.

Anemia has also been increasingly recognized as a prognostic factor in acute coronary syndromes. Anemia is linked to adverse clinical outcomes through mechanisms involving reduced oxygen delivery to tissues and elevated myocardial stress [9]. These pathways, including increased cardiac workload, contribute to poor prognosis in cardiac patients. In addition [24], a large cohort of ACS patients demonstrated that baseline anemia is highly connected to an increased mortality and major adverse cardiovascular events. These observations are in agreement with the present findings.

With regard to revascularization strategies, randomized controlled trials such as the COMPLETE trial [10] and the PRAMI trial [25] have demonstrated that complete revascularization significantly reduces long-term major adverse cardiovascular events. These findings have been reinforced by meta-analyses [26,27]. More recently, the CvLPRIT trial [28] also showed improved outcomes with complete revascularization during index hospitalization. However, as observed in the present study, the effect on early in-hospital mortality remains inconsistent, likely reflecting differences in study design, patient selection, and statistical power for short-term endpoints.

Overall, the present study confirms and extends previous findings by demonstrating that a combination of echocardiographic parameters (LVEF, IMR) and laboratory markers (hemoglobin, renal function) provides robust prognostic information in the early phase of STEMI. Compared with prior studies that often focused on individual predictors, the current analysis emphasizes the additive value of integrating multiple domains of patient assessment for improved early risk stratification.

### 4.4. Clinical Implications

The results of this study have several clinical implications. First, echocardiographic assessment of left ventricular function and mitral regurgitation should be routinely performed early after STEMI in order to identify patients at high risk of adverse outcomes. Second, laboratory parameters such as hemoglobin levels and renal function markers may provide additional prognostic information and can be easily incorporated into risk stratification models.

Early recognition of high-risk patients may allow clinicians to implement closer monitoring, more aggressive medical therapy, and optimized heart failure management. Furthermore, integrating echocardiographic findings with clinical and laboratory variables may improve risk prediction models and guide individualized treatment strategies in patients with STEMI.

Although complete revascularization was associated with improved crude outcomes, its lack of independent association with in-hospital mortality suggests that early prognosis is primarily driven by myocardial injury severity and systemic factors.

In patients with early left ventricular dysfunction after STEMI, risk stratification has important implications for arrhythmic risk and sudden cardiac death prevention. Recent evidence suggests that multiparametric assessment may guide the appropriate use of wearable cardioverter–defibrillators, helping to avoid unnecessary device implantation while identifying high-risk individuals [29].

From a secondary prevention perspective, early identification of high-risk patients may facilitate the implementation of comprehensive therapeutic strategies, including simplified approaches such as the polypill, which has been shown to improve adherence and cardiovascular outcomes [30].

In patients with reduced LVEF, early initiation of guideline-directed medical therapy is essential. Emerging evidence supports the use of novel agents such as vericiguat in selected patients with worsening heart failure [31]. Furthermore, sodium–glucose transporter 2 (SGLT2) inhibitors have demonstrated benefits in reducing cardiovascular mortality and may contribute to lowering the risk of sudden cardiac death in this population [32].

Sex differences in STEMI presentation and outcomes are increasingly recognized. Although female sex represented a substantial proportion of the study population, it was not identified as an independent predictor in the present analysis. This may reflect limited statistical power or confounding by other clinical variables. Recent evidence highlights important sex-related differences in myocardial injury patterns and prognosis, underscoring the need for sex-specific analyses in future studies [33].

### 4.5. Strengths of the Study

The present study has several strengths. The analysis included a relatively large cohort of patients with STEMI who were treated with early coronary revascularization, which reflects contemporary clinical practice. Additionally, the dataset included detailed clinical, laboratory, and echocardiographic parameters, allowing a comprehensive evaluation of potential predictors of adverse outcomes.

The strength of the study is the use of multivariable logistic regression analysis to identify independent predictors of mortality, as well as the use of graphical representations such as forest plots and ROC curves to illustrate the performance of the prediction models.

### 4.6. Study Limitations

Several limitations of the present study should be acknowledged. First, the analysis was observational in nature, which limits the ability to establish causal relationships between predictors and outcomes. Second, the data were derived from a single dataset and external validation in independent cohorts would be necessary to confirm the generalizability of the findings.

In addition, certain potentially relevant clinical variables, such as detailed angiographic characteristics, infarct size markers or long-term follow-up data, were not included in the analysis. Important clinical and angiographic variables, including Killip class, cardiogenic shock, blood pressure, heart rate, infarct-related artery, and TIMI flow grades, were not available in the present dataset. Therefore, the multivariable model may be under-adjusted, and residual confounding cannot be excluded. Biomarkers reflecting infarct size, such as peak troponin or CK-MB levels, were not available in the dataset and may represent an important source of residual confounding. Although infarct localization was recorded, it was not included in the final multivariable model. Its effects may be indirectly captured by variables such as LVEF and IMR.

Given the relatively limited number of in-hospital deaths (*n* = 48), the number of predictors included in the multivariable model may increase the risk of overfitting.

Internal validation techniques such as bootstrapping were not performed and should be considered in future studies.

In addition, no formal statistical comparison between ROC curves (e.g., DeLong test) was performed.

Additionally, the definition of complete revascularization included procedures performed during the index hospitalization, which may introduce immortal-time bias. Patients who survived longer were more likely to undergo staged procedures and thus be classified as completely revascularized. Therefore, the association between revascularization strategy and in-hospital mortality should be interpreted with caution.

## 5. Conclusions

In patients presenting with STEMI and treated with early coronary revascularization, reduced left ventricular ejection fraction, moderate or severe IMR, anemia and impaired renal function were independently associated with in-hospital mortality. These findings emphasize the importance of integrating echocardiographic and laboratory parameters into early risk stratification models. Complete revascularization was associated with improved unadjusted outcomes but was not an independent predictor of in-hospital mortality after adjustment. The identification of high-risk patients may facilitate targeted management strategies and potentially improve clinical outcomes.

## Figures and Tables

**Table 1:** Baseline characteristics of the study population.

Variable	Value
Number of patients	512
Age, years (mean ± SD)	62.0 ± 12.1
Female sex	164 (32.0%)
Smoking	213 (41.6%)
Diabetes mellitus	140 (27.3%)
HTA	340 (66.4%)
Obesity	49 (9.6%)
Dyslipidemia (LDL > 130 mg/dL)	162 (31.6%)
Anterior STEMI	237 (46.3%)
Inferior STEMI	268 (52.3%)
Lateral STEMI	62 (12.1%)
Posterior STEMI	95 (18.6%)
Right ventricular infarction	47 (9.2%)
Complete revascularization	388 (75.8%)

Note: HTA = arterial hypertension; LDL = low-density lipoprotein cholesterol; RV infarction = right ventricular infarction. Categories are not mutually exclusive, and some patients had involvement of multiple myocardial territories. Percentages may exceed 100% due to overlapping infarct territories.

**Table 2:** Clinical outcomes.

Outcome	Number (%)
In-hospital mortality	48 (9.4%)
LVEF < 40%	134 (26.2%)
Moderate/severe IMR	56 (10.9%)

Note: LVEF = left ventricular ejection fraction; LVEF < 40% indicates significant left ventricular systolic dysfunction. IMR represents moderate or severe IMR assessed by Doppler echocardiography.

**Table 3:** Outcomes according to revascularization strategy. Comparison of in-hospital outcomes between patients undergoing complete versus incomplete coronary revascularization.

Outcome	Complete Revascularization	Incomplete Revascularization	*p*-Value
In-hospital mortality	27/388 (7.0%)	21/124 (16.9%)	0.002
LVEF < 40%	84/388 (21.6%)	50/124 (40.3%)	<0.001
Moderate/severe IMR	36/388 (9.3%)	20/124 (16.1%)	0.046

Note: LVEF = left ventricular ejection fraction; complete revascularization was defined as treatment of all significant coronary lesions during the index hospitalization. Values are expressed as percentages. *p*-values were calculated using chi-square test.

**Table 4:** Laboratory and echocardiographic parameters.

Variable	Mean ± SD
LVEF (%)	43.4 ± 9.7
Hemoglobin (g/dL)	13.5 ± 1.7
Creatinine (mg/dL)	1.19 ± 0.47
GFR (mL/min/1.73 m^2^)	76.8 ± 23.4
WBC (10^3^/µL)	11.2 ± 3.8
Neutrophils (10^3^/µL)	8.2 ± 3.4
Lymphocytes (10^3^/µL)	1.9 ± 0.8
RDW (%)	13.6 ± 1.2
MPV (fL)	10.7 ± 1.2
Platelets (10^3^/µL)	234 ± 64

Note: WBC = white blood cell count; RDW = red cell distribution width; MPV = mean platelet volume; GFR = estimated glomerular filtration rate.

**Table 5:** Multivariable logistic regression predicting in-hospital mortality.

Variable	Odds Ratio (95% CI)	*p*-Value
LVEF < 40%	5.72 (2.77–11.80)	<0.001
Moderate/severe IMR	2.61 (1.14–5.97)	0.023
Hemoglobin (per 1 g/dL increase)	0.74 (0.61–0.91)	0.003
GFR (per 1 mL/min/1.73 m^2^ increase)	0.96 (0.95–0.98)	<0.001
Complete revascularization (1 vs. 0)	0.65 (0.31–1.34)	0.240

Note: CI = confidence interval; LVEF = left ventricular ejection fraction; GFR = estimated glomerular filtration rate. Odds ratios greater than 1 indicate an increased risk of in-hospital mortality, while odds ratios lower than 1 indicate a protective association.

## Data Availability

The raw data supporting the conclusions of this article will be made available by the authors upon request. The original contributions presented in this study are included in the article. Further inquiries can be directed to the corresponding author.
